# Occupational Safety and Health with Technological Developments in Livestock Farms: A Literature Review

**DOI:** 10.3390/ijerph192416440

**Published:** 2022-12-08

**Authors:** Marie A. Hayden, Menekse S. Barim, Darlene L. Weaver, K. C. Elliott, Michael A. Flynn, Jennifer M. Lincoln

**Affiliations:** 1 Division of Field Studies and Engineering, National Institute for Occupational Safety and Health, Cincinnati, OH 45213, USA; 2 Division of Safety Research, National Institute for Occupational Safety and Health, Morgantown, WV 26505, USA; 3 Office of the Director, National Institute for Occupational Safety and Health, Anchorage, AK 99508, USA; 4 Division of Science Integration, National Institute for Occupational Safety and Health, Cincinnati, OH 45226, USA; 5 Office of the Director, National Institute for Occupational Safety and Health, Cincinnati, OH 45213, USA

**Keywords:** agriculture livestock, robotics, sensors, computer vision, artificial intelligence, occupational safety and health

## Abstract

In recent decades, there have been considerable technological developments in the agriculture sector to automate manual processes for many factors, including increased production demand and in response to labor shortages/costs. We conducted a review of the literature to summarize the key advances from installing emerging technology and studies on robotics and automation to improve agricultural practices. The main objective of this review was to survey the scientific literature to identify the uses of these new technologies in agricultural practices focusing on new or reduced occupational safety risks affecting agriculture workers. We screened 3248 articles with the following criteria: (1) relevance of the title and abstract with occupational safety and health; (2) agriculture technologies/applications that were available in the United States; (3) written in English; and (4) published 2015–2020. We found 624 articles on crops and harvesting and 80 articles on livestock farming related to robotics and automated systems. Within livestock farming, most (78%) articles identified were related to dairy farms, and 56% of the articles indicated these farms were using robotics routinely. However, our review revealed gaps in how the technology has been evaluated to show the benefits or potential hazards to the safety and well-being of livestock owners/operators and workers.

## 1. Introduction

Agriculture accounted for about 1.4% of the U.S. labor force in 2020, with approximately 2.6 million people employed in full- and part-time jobs [1]. The agriculture sector ranks among the most hazardous industries worldwide, with high rates of occupational fatalities, injuries, and illnesses [2,3]. In 2020, U.S. agriculture, forestry, fishing, and hunting industries had a rate of 21.5 fatal injuries per 100,000 full-time equivalent workers, which was roughly six times the average rate of 3.4 for all private industries [4]. Likewise, the 2020 nonfatal occupational injury incident rate for agriculture, forestry, fishing, and hunting averaged 4.6 per 100 full-time workers, which was higher than the average of 2.9 for all industries [5]. According to 2020 Bureau of Labor Statistics (BLS) data, agricultural workers suffered 18,750 nonfatal occupational injuries and illnesses that resulted in days away from work, but this number excludes injuries on farms with fewer than 11 workers [6]. U.S. Agriculture, forestry, and fishing industry workers are exempt from some worker safety and health policies, which may affect the reporting of nonfatal injuries [7,8,9,10]. Furthermore, nonfatal events or exposures that made workers ill or injured included contact with objects and equipment (4710 cases), falls, slips, trips (4750 cases), and overexertion and bodily reaction (3060 cases) [11].

Agriculture workers are exposed to a variety of work-related hazards. In traditional agriculture, those who work on farms and ranches are engaged in labor-intensive processes such as dairy operations (milking) [12], feeding animals, and monitoring animal behaviors (e.g., detecting lameness, disease, and management/housing farms) [13]. Along with labor-intensive processes from cleaning the livestock pens or stalls, workers inhale bioproduct gases created from the manure [14]. In recent decades, considerable technological developments, including the internet of things (IoT), sensors, robotics, drones, and artificial intelligence (AI), have been transforming the agriculture sector. These new technological developments have led to what is commonly known as precision agriculture to assist with managing and optimizing farm health and productivity. Data gained from precision agriculture are utilized to reduce and target inputs in more effective ways [15]. Each technology has different advantages. For example, with sensory data, farmers can collect information much easier without needing to interact with animals. The IoT helps farmers to easily analyze data on weather, temperature, moisture, and prices and provide insights into how to optimize yield, improve planning on location monitoring, make smarter decisions about the level of resources needed, and determine when and where to distribute those resources to minimize waste and increase yields [16]. In general, robotic systems are in use and under development for autonomously monitoring livestock and collecting field data continuously without human–animal interaction [15,16].

New technological innovations impact both workplaces and workforces, and these developments are poised to take over some of the physical labor tasks of animal farming. However, the adoption of these technologies for livestock varies widely across technologies, animal species, and areas of application. These ongoing changes in the workplace, work, and workforce have continued to shape the Future of Work [17]. While such technological developments have offered many opportunities, they have also posed challenges. One of the research goals of the NIOSH Future of Work Initiative is to mitigate worker safety and health challenges and leverage opportunities associated with the objective of evaluating the benefits and risks of robotics [18]. Another is to evaluate the impact of innovative and emerging technologies on worker well-being. With respect to those two NIOSH Future of Work Initiative goals, we conducted a review of the literature to summarize the key advances from installing emerging technology and research studies on robotics and automation to improve agricultural practices. The main objective of this review was to survey the scientific literature to identify the uses of these new developments in agricultural practices focusing on the occupational safety and health (OSH) of the agriculture workers.

## 2. Materials and Methods

We conducted a pilot search to find terms that would reflect emerging technologies (Table 1 search string 1) and the type of agricultural products found in crop and livestock production (Table 1 search string 2). The team then worked with a Center for Disease Control and Prevention Librarian to finalize our search terms and determine appropriate databases. Relevant articles from the years 2015–2020 were searched from five databases: Scopus, IEEExplore, Environmental Science Collection, CAB Abstracts, and Agricultural Science Collection (ProQuest, Agricola). Specifically, different combinations of search terms (Table 1) were used with logical operators ‘AND’ and ‘OR’.

**Figure 1:**
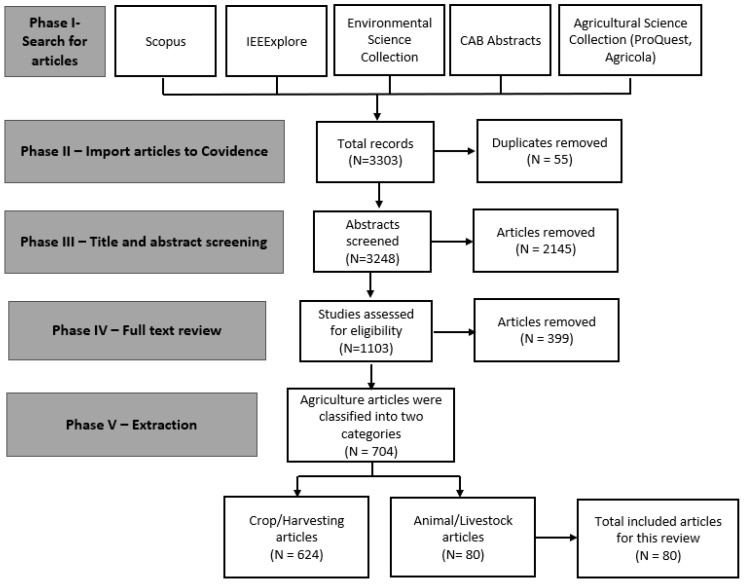
Flow chart of articles selection process.

The initial search of the literature identified more than 3000 articles. Titles and abstracts of these articles were screened according to the following criteria: (1) relevance of the title and abstract to the topic of OSH; (2) agriculture technologies/applications that were available in the United States; (3) written in English; and (4) published 2015 to 2020. Five independent reviewers performed the screening using Covidence [19], a literature review management software that requires two reviewers to vote on articles in each phase (Figure 1). Articles that had the same decision by two reviewers would be moved to the next step or removed. Reviewers would remove articles in agriculture sub-industries for forestry or aquaculture. Articles in which one reviewer suggested inclusion, whereas the other suggested discarding, were screened by a third reviewer for a final decision. If the third reviewer could not resolve the conflict, it was decided by consensus among the three reviewers. As crop production job tasks and technology vary greatly from those in livestock production, articles were then separated by “crop/harvesting” (624) and “animals/livestock” (80) during phase five data extraction. Articles focused on crop/harvesting and general information on livestock were included in data extraction for crop/harvesting. Based on the above criteria, 80 articles were then selected for the extraction phase, specifically for livestock (Figure 1). Relevant data were then extracted and subsequently categorized by author, year, purpose of the study, type of technology, job task, and potential benefits or hazards to farmers or farm workers.

## 3. Results

### 3.1. Type of Technology Used in Livestock

Of the 80 articles on livestock farming selected for the full review process, 64 articles (80%) focused on dairy (cattle, calves, sheep, and goats) activities, 10 articles (12.5%) on pork (pigs and piglets), 3 articles (3.75%) on poultry (chicken), and 3 articles (3.75%) included multiple farm animals such as cattle and pork. Fifty articles (63%) focused on the reason and application to apply technology for the management of animal health (e.g., monitoring cattle health, disease detection, lameness, and animal behavior), and the remaining 27 articles (37%) focused on technology features, productivity, or quality. Two articles focused on occupational exposure, and one article reviewed the relationship between humans and animals with emerging technologies. Thirty-five articles (44%) reported on the use of emerging technologies such as sensors, computer vision, and AI; thirty-five articles (44%) discussed the use and productivity of robotics and advanced automated systems; and 10 articles (12%) combined sensors and robotics. More than half of the 80 articles were related to automated robotic equipment in the dairy industry. These systems were primarily developed to reduce manual milking and in response to labor shortages/costs. Dairy studies compared different automatic milking systems (AMS) and automatic feeding systems (AFS) to see which model had more advantages in efficiency and animal welfare. The main purpose of dairy studies with AMS was to test milking time, time in AMS, milk yield, milk flow, and milking intervals [20].

The articles describing emerging technologies such as sensors, computer vision, and AI-focused more on animal welfare as a justification to adopt these devices rather than the impact of these devices on health and safety for the farmer and/or worker. Farmers use this new technology to monitor animal behaviors visually with IoT-based computer vision techniques [21], infrared images to measure eye and cheek temperatures [22], and visual scans of foraging activities to study aging [23]. Sound based technology includes collecting sound data to propose an algorithm to detect bovine respiratory disease [24] and classify sound to understand distress vocalizations in poultry, pork, and beef [25].

Seventeen (21%) of 80 articles (Table 2), directly evaluated occupational exposure or indirectly addressed or discussed the central focus of our review, i.e., the impact of technology on worker safety, health, or well-being when handling or feeding animals or cleaning and maintaining indoor barn environment. Specifically, Basinas et al. and Böhlandt et al. studied the impact of technology on occupational health exposures and found positive outcomes of reduced exposure to dust and endotoxins [26] and cow hair allergens [27] with the automated system compared to manual. An additional 15 (19%) articles considered topics such as labor savings and mental workload affecting farm productivity that can also affect worker health and well-being; however, the articles did not specifically study the impact on the safety and health of the workers. The majority described positive outcomes, while only a few articles noted some potential safety concerns. The following paragraphs describe the studies which pertain to human safety and health regarding new technologic advancements.

### 3.2. Occupational Exposure

Of the two articles that evaluated occupational exposure, Böhlandt et al. [27] measured the concentration of cow hair allergen airborne and settled dust in the work areas and private living spaces. They collected samples using three different methods at 12 farms with established AMS and eight farms with conventional milking systems. Sampling took place within the milking stables, changing rooms, living rooms, and mattresses for all farms. The farms with AMS had samples taken from a computer room that monitors the AMS. Results showed that the concentration of cow hair allergen decreased from the milking stables to the private living spaces. In addition, allergen concentrations were lower in the private living spaces of farms using AMS compared to farms using conventional milking systems. The authors suggested that this difference could be due to the AMS having a separate computer room from the milking stables, which created a barrier decreasing close animal contact and reducing exposure time for allergen transfer during milking [27].

The second study, Basinas et al. [26], measured exposure to dust, endotoxin, and total volatile organic compounds (VOCs) at seven different dairy farms with three types of feeding systems. Three farms used semi-automated feeding systems, three operated manually, and one used a loft feeding system (which requires a worker to transfer feed into the hopper manually). Results from the study indicated the difference in endotoxin exposure between the type of feeding material and suggested inhaled endotoxins averaged 42% lower with semi-automatic vs. manual feeding [26]; however, the loft feeding system was not included in the comparison due to the small sample size.

### 3.3. Occupational Health and Wellbeing

Few studies tested the adoption process of AMS, AFS, or other automation in small and midsize farms, but overall the studies with large herd sizes found positive outcomes for workers, decreased labor costs, and/or increased productivity/efficiency. Schewe and Stuart [32] found positive outcomes for family and non-family labor, animal welfare, the environment, and financial resiliency. Butler and Holloway [30] described how implementing AMS changed farmers’ social status and work-life balance. Rodenburg [29] reviewed studies to show how specific factors from herd management and facility design contributed to the effects of labor, the performance of automatic robots, and animal health. Their review found two studies [41,42] reported around 20% labor savings, one study [43] with no significant labor savings due to increasing herd size, and two studies [44,45] reported a new labor demand from fetching cattle that did not voluntarily go to the robotic milking system [29]. The average for fetching cows from 41 AMS farms was 8.1%/day [44] and 14.6%/day from 43 AMS farms varying in fetching frequency due to barn design [45]. Rodenburg and House found reasons why workers had to fetch cattle were due to the cow’s inexperienced with the AMS process, issues with the AMS teat placement, visible lameness or health issues with the cow, and other cases had no identifiable reason [45]. Tse et al. [46] surveyed farms to evaluate the impact of AMS on reducing labor and increasing milk production. Their survey results taken from 214 farms with AMS found a 20% decrease in the number of employees to care for an assigned herd size from a mean of 2.5 employees to 2.0 employees [46]. Tse et al. reported two different AMS models reduced the milking time by 2.5 h/day and 3.4 h/day. They also reported a median of three cows per robot that required the worker to go and fetch the cow [46]. Salfer et al. [13] surveyed workers and collected data at fifty-four dairy farms that implemented AMS. Salfer et al. [13] noticed labor efficiency with larger farms before the AMS averaged 96 cows/full time employee compared to now having some cases of handling three to five cattle per day that failed to voluntarily go towards the AMS. Their survey found farmers’ reasons for transitioning to AMS were the benefits of less labor from repetitive milking (60%), improved lifestyle allowing for additional free time (55%), and human health (28%) [13]. Hansen [31] completed 19 interviews with Norwegian dairy farmers to explore motivators for adopting AMS and consequences for farmers’ lifestyle and management. Their results described the farms sampled were 2.5 times the size of average farms because AMS led to expanding their production. They noted an increase in flexibility as the greatest advantage but suggested it came at a disadvantage because farmers felt they were never off duty, such as responding to an alert from a robot in the nighttime [31].

Tangorra and Calcante [33] measured energy consumption and technical-economic analysis of AFS. In this study, they calculated costs for preparing and distributing a total mixed ration (TMR) with AFS and compared it with the conventional feeding system (tractor + TMR wagon). They found a 97% reduction in energy consumption and 79% labor cost savings [33]. Borso et al. [34] found energy consumption for conventional feeding systems used 94.00 kilowatts (kWh) per day compared to the AFS, which used 68.05 kWh per day, and they found a reduction in labor requirements with an AFS at 1.02 h per day compared to the conventional feeding system at 2.5 h per day. The study revealed a strong reduction of energy consumption by 97% and manual labor by 79% when adopting an AFS in comparison to a conventional feeding system. Both of these reductions contributed to reducing the daily cost of feeding TMR by up to 33% [34]. Hennings et al. [35] analyzed European Union (EU) milk operating costs from 2007 to 2014, and they found that a well-implemented feeding strategy can limit treatment and veterinary costs as well as production losses.

Chiumenti et al. [38] focused on evaluating the performance of a robotic scraper based on gaseous emissions from two types of flooring. Installation and use of robotic scrapers were motivated by the reduced manual labor and the more frequent removal of manure that affects the animal and farmer’s health due to the produced gases from fermentation. There were reductions found in the emission rates for methane (CH_4_), nitrous oxide (N_2_O), and carbon dioxide (CO_2_) after cleaning on both concrete and rubber flooring. They did find an increased emission of ammonia (NH_3_) with rubber flooring after cleaning compared to dirty floors [38]. Drach et al. [36] reported that the use of an automated herding system functioned to move cattle toward the milking system had increased milk yield and decreased 80% in work time compared to manual fetching. Winnicki and Jugowar [28] tested the transition of cows from the shed with a stanchion-tied maintenance system and a pipeline milking with a cubicle maintenance system. They found out that this cubicle maintenance system increases work productivity, improves the comfort of living for cows, improves the quality of milk, increases milk yield, and improves working conditions [28]. Vroegindeweij et al. [37] evaluated the performance of a prototype autonomous mobile robot for the purpose of collecting poultry house eggs found on the floor. This was motivated to reduce the farmer’s task of manually collecting eggs on the floor. Their results focused primarily on the operation to pick up 300 eggs and the mobile robot successfully collected 46% without damage. This study’s authors concluded that improving navigation and obstacle avoidance algorithms would improve egg collection efficiency and would be necessary for commercial feasibility [37]. Lee et al. [21] monitored an IoT-based large-scale smart farm to detect undergrown pigs in group-housed pig rooms with deep-learning-based computer vision techniques. They found that this method should be considered as an aid (e.g., sending alarms) rather than as a replacement for farm workers. Deokar et al. [40] developed an IoT-based smart farm system that notifies farmers on levels of feed filling system, water filling system, biogas exhaust system, and fire detecting systems. Their study is ongoing but discussed that continuous automated monitoring of physical parameters such as water and feeding levels would allow increased work flexibility and ease of workload for farmers [40].

Furthermore, Hostiou et al. [39] reviewed studies observing changes in farmers’ tasks with respect to workload and animal–farmer relationships following the implementation of different sensor and robotic technologies. One task was the change from manually milking cattle to the farmer monitoring the AMS via a computer. Hostiou et al. [39] noted that AMS created flexibility of time for the farmers but also created new tasks, such as fetching cattle that had not gone to the milking station. Hostiou et al. also discussed some of the advantages of the automated process with sensors, such as the advantage of early disease detection, monitoring the reproduction cycle, or feeding intake. However, they also noted the amount of information generated could increase the farmer’s mental workload due to the increased need to understand and select relevant information as a part of the decision-making process. Moreover, Hostiou et al. [39] found automated process changes reduced positive physical interactions such as manual milking but did not reduce manual negative interactions such as vaccinations, castration, and trimming, which may impact the overall human/animal relationship experience. Likewise, des Roches et al. [47] assessed the human-animal relationship at 118 farms using a milking parlor (manual) or AMS by observing the cow’s avoidance reaction at the feeding rack and surveyed the farmers’ attitude towards the cow. Their results showed farms with AMS had a lower portion of cattle avoidance when the observer approached cattle at the feeding rack compared to the conventional farms. The study concluded that the relationships between the worker and cattle varied by the management process and attitude of the farmer. Nothing from their study found the AMS system would be a factor that would affect the relationship positively or negatively between the worker and animal [47].

## 4. Discussion

The introduction of new technologies into the workplace can have significant impacts, both positive and negative, on the health, safety, and well-being of workers [17]. Despite this, the results of our review found that while some studies acknowledged the potential impact on farmer safety and health, most studies largely ignored the impact of technology on worker safety by focusing instead on animal health (63%) or improving equipment features (34%). On an encouraging note, in the limited number of papers that directly or indirectly addressed OSH, there was a notable breadth of topics. Studies not only addressed traditional OSH concerns such as biological exposures (e.g., dust and cow hair allergens) but also addressed issues related to work-life balance, economic impact, mental workload from the alarm systems, and the emotional impact of changes to farmer-animal relationships resulting from the introduction of technology.

The limited number of studies that directly or indirectly discussed topics related to farmer/farmworker well-being used established robotic and sensor technology compared to the emerging sensors, computer vision, and machine/deep learning technologies which are becoming popular in agriculture. The studies within our review mentioned applying these emerging technologies to monitor animal welfare but only discussed improvement in precision and accuracy detection with these new technologies. There are other research studies that focus on animal welfare discussed in Buller et al.’s review of the literature [48]. These studies within our review neither tested the effect of these technologies on animal welfare nor provided any evidence of how these technologies impacted the safety and health of the farmers and/or farm workers. A similar review of the literature for the dairy sector found a similar gap, with only 16 studies out of 343 (5%) assessing the impact of interventions for workers’ safety and health, and only one of those 16 studies examined farmer’s and farm workers’ mental well-being [49].

There were only two articles in this literature review that discussed changes in the human and animal relationship dynamic due to incorporating automated technology [39,47]. Both discussed the relationship changes associated with farm management and did not evaluate the impact the relationship may have on the safety and health of farmers and farm workers.

Only two studies quantified OSH respiratory exposures [26,27] and were limited to the dairy sector. Our review did not find any assessment on traditional OSH exposures or events such as the ergonomic impact on musculoskeletal disorders, or vibration, noise, heat and cold stress, slips, trips or falls, and cuts or lacerations for the farmer and/or farm worker with these emerging technologies. The indirect OSH topics of reduced labor and flexibility were focused more on the benefits or concerns related to production instead of worker well-being. Although some studies discussed the positive and negative impacts of adopting new technology, none directly evaluated those impacts on worker well-being, such as worker fatigue, stress, or mental health with the new technology.

As for limitations, this review only focused on robotic equipment and emerging technologies with artificial intelligence in livestock production operations and excluded meat processing and crop production operations. When reviewing these dairy studies, we did not include the differences created between the barn designs, such as free flow traffic versus guided flow [50]. Limiting articles to only English and selecting equipment used in the United States created the potential for bias. This may create a loss of technology that could impact the safety and health of the workers that was not captured in this review.

## 5. Conclusions

New technological developments to automate and streamline agricultural activities may improve the safety and health of agriculture workers/farmers. However, there are notable gaps in research for comparing how the technology can benefit the workers or mitigate safety and health concerns associated with the technology. Most of the technology discussed in these studies seems to decrease physical labor and/or hazard exposure of workers. At the same time, most studies only discussed the application of the technology itself and potential productivity increases and neither tested nor discussed the worker safety and health advantages/disadvantages of using these technologies or their potential contribution to technological job displacement. For livestock farming, we identified that robotic technologies are well-established compared to sensors, computer vision, and artificial intelligence, which are slowly gaining adoption in crop agriculture. Likewise, we observed how these robotic technologies could automate repetitive tasks; however, more research on the potential safety and health effects for farmers and farm workers is needed. Areas of future research should consider how to assess barriers to the adoption of new technology as well as potential unintended consequences automated technology may have on the human/animal relationship, which could impact worker safety and well-being. Future research should also address the potential differential impact of these technologies across the workforce and within the industry and how their adoption may aggravate or ameliorate occupational health inequities [51]. For example, technology that reduces the need for manual labor can at once result in increased profits for farmers while resulting in job loss for farm workers. Similarly, the initial capital investment to adopt some technologies may increase production and make work safer on larger farms but could simultaneously place smaller ones at a competitive disadvantage which could negatively impact the well-being of these farmers.

## Figures and Tables

**Table 1:** Search strings used to identify articles.

Search String	Search Terms
1	‘Robot*’ OR ‘Drone*’ OR ‘Artificial intelligence’ OR ‘Agbot*’ OR ‘Agrobot’ OR ‘Autonomous’ OR ‘Mobile technology adoption’ OR ‘Smart agriculture’ OR ‘Sensors’ OR ‘Smart farming’ OR ‘Technology adoption’ OR ‘Smart machines’ OR ‘Automated harvesting’ OR ‘(Automated) Agricultural machinery’ OR ‘Human-Robot collaboration’ OR ‘Robot’
2	‘Soft gripping’ OR ‘Picking’ AND (‘Agriculture’ OR ‘Agricultural’ OR ‘Poultry production’ OR ‘Egg production’ OR ‘Swine production’ OR ‘Beef production’ OR ‘Livestock’ OR ‘Crop production’ OR ‘Farming’ OR ‘Farmer*’ OR ‘Farm-hand*’ OR ‘Farmhand*’ OR ‘Farm work*’ OR ‘Ranch*’ OR ‘Orchard’ OR ‘Fruit’ OR ‘Vegetable’ OR ‘Harvest’)
3	NOT exp animals
4	NOT exp humans

**Table 2:** Studies that address or discuss the impact of technology on worker safety and health.

Equipment Category	Date	Author	Geographic Locations	Purpose of the Study	Safety and Health Topics
Robotics (Milking)	2018	Salfer et al. [13]	Upper West, United States	Housing, management characteristics, and factors affecting animal health hygiene	Indirectly-labor
Robotics (Milking)	2018	Winnicki et al. [28]	Poland	Comparing effects of milking on conventional farms converted to an automatic farm	Indirectly-labor
Robotics (Milking)	2017	Rodenburg et al. [29]	Ireland	Risk factors with production processes associated with AMS traffic flow	Indirectly-labor and well-being
Robotics (Milking)	2016	Butler et al. [30]	United Kingdom	Restructuring work practices on dairy farms with AMS	Indirectly-well-being
**Robotics** **(Milking)**	**2016**	**Böhlandt et al.** [27]	**South Germany**	**Farmer’s concentration exposure to cow hair allergen with automatic vs. conventional milking systems**	**Directly-occupational exposure**
Robotics (Milking)	2015	Hansen, B.G. [31]	Norway	Farmers’ reasoning and perception on adoption of AMS	Indirectly-well-being
Robotics (Milking)	2015	Schewe et al. [32]	United States Midwest, the Netherlands, and Denmark	Surveying farmers for different factors on why they adopted AMS	Indirectly-well-being
Robotics (Feeder)	2018	Tangorra et al. [33]	Italy	Assessing electrical consumption of automatic equipment on farm	Indirectly-labor
**Robotics (Feeder)**	**2017**	**Basinas et al.** [26]	**South West Ireland**	**Measuring exposure of dust, endotoxin, and VOCs during feeding and milking process with manual and semi-automatic feeding systems**	**Directly-occupational exposure**
Robotics (Feeder)	2017	Borso et al. [34]	Northeast Italy	Structure modification to adapt automatic feeder	Indirectly-labor
Robotics (Feeder)	2016	Hennings, C. [35]	Europe	Economic impact of the automatic feeders on animal welfare-nutritional intake	Indirectly- labor
Robotics (Automated gates)	2017	Drach et al. [36]	Israel	Herding cattle to the robotic milking station	Indirectly-labor
Robotics (Cleaner-collects eggs)	2018	Vroegindeweij et al. [37]	Europe	Assessment of autonomous mobile robot for collecting floor eggs	Indirectly-labor
Robotics (Cleaner-manure)	2018	Chiumenti et al. [38]	North-Eastern Italy	Exposure of gaseous emissions and environmental management between different surfaces	Indirectly-occupational exposure
**Robotics (Sensors)**	**2017**	**Hostiou et al.** [39]	**No regional location provided**	**Relationship between farmers and animals using automated devices (review article)**	**Directly-labor and well-being**
Robotics (Sensors and platform)	2018	Deokar et al. [40]	No regional location provided	Monitoring the animal health and environment on the farms	Indirectly-labor and well-being
Machine/Deep Learning	2019	Lee et al. [21]	South Korea	Monitoring swine (pigs) for quality of growth	Indirectly-well-being

## Data Availability

The data that support the findings of this study are available from the corresponding author upon reasonable request.
